# Mechanisms of σ^54^-Dependent Transcription Initiation and Regulation

**DOI:** 10.1016/j.jmb.2019.04.022

**Published:** 2019-09-20

**Authors:** Amy E. Danson, Milija Jovanovic, Martin Buck, Xiaodong Zhang

**Affiliations:** 1 Section of Structural Biology, Department of Medicine, Imperial College London, London SW7 2AZ, UK; 2 Department of Life Sciences, Imperial College London, London SW7 2AZ, UK

**Keywords:** bEBPs, bacterial enhancer-binding proteins, ELH, extra-long helix, HTH, helix-turn-helix, RNAP, RNA polymerase, RPc, closed promoter complex, RPi, intermediate promoter complex, RPip, intermediate complex with partially loaded DNA, RPo, open promoter complex, RPitc, initial transcribing complex, T-, template strand, RNA polymerase, sigma factors, bacterial enhancer-binding proteins, transcription bubble, transcription regulation

## Abstract

Cellular RNA polymerase is a multi-subunit macromolecular assembly responsible for gene transcription, a highly regulated process conserved from bacteria to humans. In bacteria, sigma factors are employed to mediate gene-specific expression in response to a variety of environmental conditions. The major variant σ factor, σ^54^, has a specific role in stress responses. Unlike σ^70^-dependent transcription, which often can spontaneously proceed to initiation, σ^54^-dependent transcription requires an additional ATPase protein for activation. As a result, structures of a number of distinct functional states during the dynamic process of transcription initiation have been captured using the σ^54^ system with both x-ray crystallography and cryo electron microscopy, furthering our understanding of σ^54^-dependent transcription initiation and DNA opening. Comparisons with σ^70^ and eukaryotic polymerases reveal unique and common features during transcription initiation.

## Highlights

• Mechanism of transcription inhibition by σ^54^
• Coupled load and unwind model for transcription initiation
• Role of bacterial enhancer-binding proteins in isomerization to the open complex
• Comparison of structural and functional differences between σ^70^ and σ^54^

## Graphical Abstract

**Figure:**
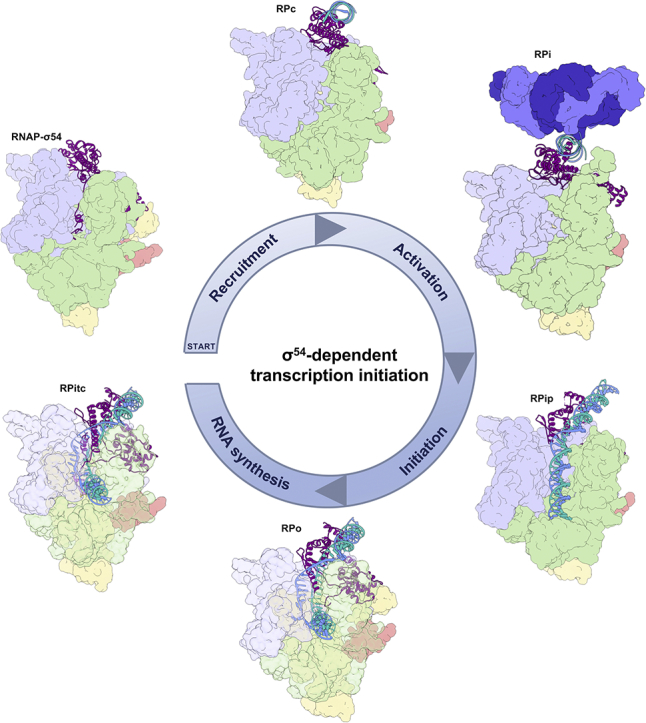

## Introduction

Transcription must be tightly controlled to ensure an adequate and efficient response to growth and environmental changes. The strict regulation of gene expression is controlled in a number of ways with bacteria relying on sigma (σ) factors as the primary regulatory factors. These factors confer gene specificity by directing RNA polymerase (RNAP) to a selective set of genes under their control. Subsequent regulations are imposed during the distinct stages of transcription, with the majority executed during initiation, the stage involving the conversion from the initially closed complex, where DNA remains double stranded and outside of RNAP, to the transcriptionally competent open complex, where the transcription bubble has opened up and the template (T-) strand has been delivered into the active centre.

Cellular RNAP is conserved from bacteria to humans with the bacterial enzyme containing the minimal core of five subunits: β, β′, two α, and ω subunit [1], [2]. A number of σ factors exist in bacteria ranging from seven in *Escherichia* and *Shigella* to 65 in *Streptomyces coelicolor* [3], [4]. σ factors can be broadly classified into two families based on their functions and mechanisms. The σ^70^ class comprises the primary σ factor (σ^70^ in *Escherichia coli*), which regulates housekeeping genes, and alternative σ factors involved in the stress response [5]. σ^70^ can be further divided into four groups, which are characterized by varying degrees of conservation in the four structural domains [6]. σ^70^ recognizes the − 35 and − 10 promoter sites (upstream from the transcription start site, TSS at + 1) and can spontaneously isomerize from a closed complex into an open promoter complex [6].

σ^54^ (or σ^N^), which is involved in a range of different stress responses, has no significant sequence similarity to σ^70^ and instead recognizes − 24 and − 12 promoter sites [7]. Unlike σ^70^, the σ^54^ closed complex (RPc) is unable to spontaneously isomerize to an open complex (RPo). Instead it requires ATP dependent activator proteins bound remotely upstream from the promoter site in order to activate transcription.

The σ^54^ activator proteins, termed bacterial enhancer-binding proteins (bEBPs), contain a conserved ATPase domain that belongs to the large ATPase associated with various cellular activities (AAA +) protein family [8]. Initially, bEBPs interact with upstream DNA (the enhancer-binding sites), approximately 100–150 base pairs (bp) upstream of the promoter region termed the upstream activating sequence [9]. bEBPs then oligomerize, often aided by nucleotide binding to the AAA + domain, and use its ATPase activity to remodel the closed complex. AAA + domains contain the highly conserved AAA-specific motifs for nucleotide binding and hydrolysis as well as the highly conserved GAFTGA motif, which was shown to be involved in interacting with σ^54^ through a direct contact [10]. Most bEBPs contain a single polypeptide chain that forms a homohexamer. As an exception two bEBPs, HrpR and HrpS from *Pseudomonas syringae* need to form a hetero-hexameric complex (HrpRS) in order to activate transcription [11]. Although HrpR and HrpS show high-sequence similarity and possess all conserved bEBP central domain regions and all characteristic motifs, HrpR and HrpS functions are highly co-dependent and are completely incapable of activating transcription on their own, suggesting individual subunits within the bEBP hexamers play unique roles during open complex formation.

**Fig. 1:**
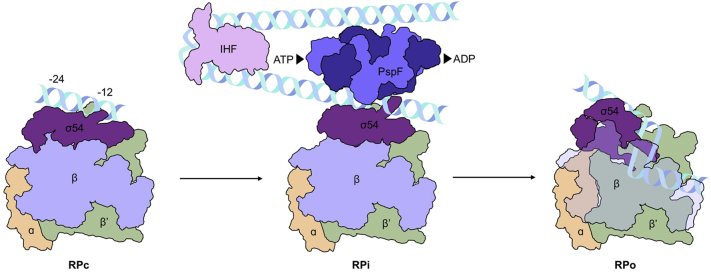
Schematic of the basic mechanism of σ^54^-mediated transcription initiation between the closed (RPc) and open (RPo) DNA complex. The β subunit of RPo is shown in transparent in order to visualize the open transcription bubble.

Most bEBPs are regulated by stress-related signals *in cis* (such as NtrC, NorR and NifA), through their N-terminal regulatory domains [10]. Like PspF, both HrpR and HrpS lack a cis-regulatory domain, and therefore, HrpRS hetero-hexameric activity is negatively regulated *in trans* by HrpV which specifically binds HrpS [12], whereas PspF is negatively regulated by PspA. As the upstream activating sequence region is not in close proximity to the promoter region, where the RNAP-σ^54^ holoenzyme awaits, DNA looping is required, which often is assisted by the integration host factor, in order to bring the bEBPs into the vicinity of the promoter (Fig. 1) [13], [14], [15].

Isomerization of closed to open promoter complexes involves opening up the double-stranded (ds) DNA into a transcription bubble in the span of 13–15 bp just upstream of the TSS (between − 12 and + 1). The T-strand is then orientated into the RNAP active site where transcription is carried out. However, until recently, the exact mechanisms involved in the initiation process were poorly understood due to the dynamic and transient nature of this process. As the RPc cannot spontaneously proceed to transcription in the absence of ATPase activity, the σ^54^ system has been particularly useful for characterising distinct functional states and has recently led to the capture of not only the closed and open complexes, but also two intermediate states, providing valuable insight into the key stages of bacterial transcription [16], [17], [18].

The σ^54^ system is a fascinating system to study the initiation process due to its unique properties in controlling transcription as well as the coupling of cellular signals to transcription initiation through the bEBPs. Furthermore, σ^54^ activators represent a unique class of AAA + proteins. A number have recently been shown to act through a sequential mechanism by threading substrates through its hexameric central pore [19], [20], [21], [22], [23]. However, it is unknown how other AAA + proteins including bEBPs, which do not actively thread substrates through their central pores, couple nucleotide activities to their functions.

Much progress has been made in the last 30 years in our understanding of molecular mechanisms of the σ^54^-dependent transcription regulation biochemically and genetically. However, unlike the σ^70^ system, which has had a number of high-resolution structures since early 2000 including the holoenzyme, the complex with fork junction DNA, and more recently the open complex and initial transcription complex [24], [25], [26], [27], [28], [29], high-resolution structures of the σ^54^ holoenzyme, and subsequent DNA complexes were only available from 2015 oownward. This review will thus focus on the recent structural data and summarize our current understanding of the molecular basis of transcription initiation in σ^54^-dependent transcription and the role of ATPase activator proteins. In particular, we will detail our mechanistic model on transcription bubble formation and DNA loading, captured recently in σ^54^-dependent intermediate states. Furthermore, here we discuss the structural features that define the unique properties of σ^54^ in comparison to σ^70^ and provide a structural basis on why σ^54^ is unable to spontaneously isomerize to the open complex.

## Molecular Basis of Transcription Inhibition by σ^54^

**Fig. 2:**
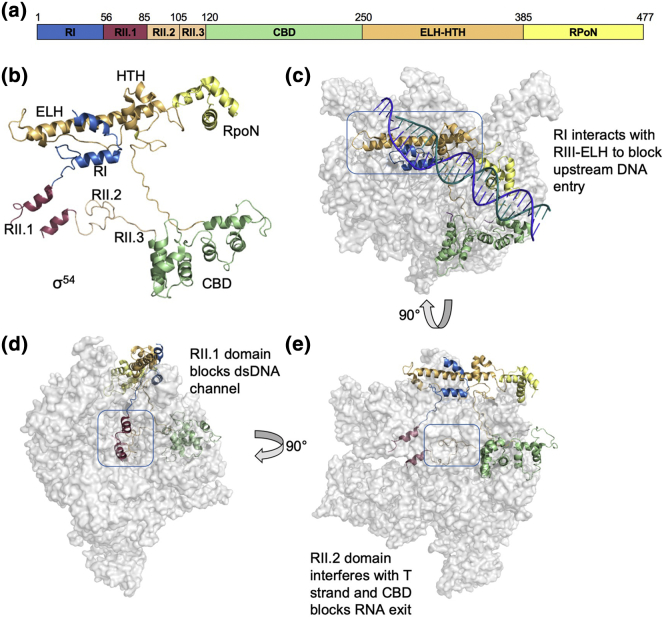
The inhibitory action of σ^54^ on RNAP. RNAP (αββ'ω) is shown in gray, with key interaction sites highlighted in blue boxes. (a) Primary structure of σ^54^. (b) Structural components of σ^54^. (c) The upstream T-strand site is blocked by the interaction between RI and RIII-ELH. (d) RII.1 occupies downstream dsDNA channel. (e) RII.2 restricts T-strand entry, while RII.3 occupies the RNA path and the CBD blocks the RNA exit channel. Images created in PyMOL v1.8.4.0 with PDB ID: 5BYH (a, c, d) and 5NSR (b).

*E. coli* σ^54^ consists of three main functional regions based on sequence alignments, denoted regions I–III (RI–III), shown in Fig. 2a and b. N-terminal RI, between residues 1–56, is responsible for the interaction with the activator proteins as well as having an inhibitory role in formation of the RPo [30]. RII, located at residues 57–120, is poorly conserved, whereas RIII contains the major RNAP binding domain, the core binding domain (CBD), as well as major DNA binding domain RpoN, which recognizes the − 24 promoter DNA (Fig. 2b and e) [31], [32], [33], [34], [35]. The crystal structure of RNAP-σ^54^ and the cryo electron microscopy (cryoEM) structure of the RNAP-σ^54^–DNA closed complex, RPc, revealed a number of structural domains (Fig. 2). RI consists of two helices forming a hook. RII can be further divided into RII.1, RII.2 and RII.3. RIII consists of the CBD followed by loops that lead to the extra-long helix (ELH), a 55-Å-long helix whose N-terminal portion forms an interacting domain with RI, whereas its C-terminal region is part of the helix-turn-helix (HTH) motif, before finally leading to the RpoN domain (Fig. 2b) [16].

The role of σ^54^ RI in transcription inhibition was first recognized through mutagenesis of the leucine and glutamine-rich patch, as these mutations permitted activator-independent transcription [36]. Four random point mutations within this region were proficient in bypassing NtrC, the bEBP required for nitrogen metabolism [36]. Interestingly, a number of other activator bypass mutants have been identified in regions outside RI, including R336A in RIII-ELH [32], [37]. These findings suggested a network of interactions within σ^54^ acted to maintain RPc and prevent its isomerization.

Earlier low-resolution cryoEM reconstructions of RNAP-σ^54^ holoenzyme identified a domain in σ^54^ capable of blocking the entrance of DNA into the RNAP active channel and this domain was hypothesized to be RI [38]. However, the detailed molecular basis of inhibition was only revealed when the 3.8-Å structure of RNAP-σ^54^ was resolved using x-ray crystallography [16]. The cryoEM structure of the RPc provides further insights into the mechanism of inhibition by σ^54^ [17].

These structures show that the interacting RI and RIII-ELH of σ^54^ stably block DNA access to the RNAP active site by occupying the β and β′ cleft (Fig. 2c), which is also too narrow to accommodate double-stranded DNA in the holoenzyme and RPc [16], [17], [38]. These structures explain how RI inhibits transcription and why mutations in or deletion of the RI or mutation of the R336 residue within the ELH domain of RIII, the specific region shown to interact with RI (Fig. 2a-b), removed the requirement for an activator protein, provided the transcription bubble is preformed [16], [32], [37].

In addition, RII and its three subdomains (RII.1–3) were shown to occupy a number of sites within the RNAP known to be essential for DNA binding (RII.1, Fig. 2d), DNA T-strand organization (RII.2, Fig. 2e), as well as the channel designated to newly synthesized RNA (RII.3), thus suggesting functional importance for this highly variable and previously unassigned region [16]. Interestingly, in the closed complex solved by cryoEM, the density is weak within this region, suggesting the dynamic nature of RII. These regions are connected by flexible linkers, thus likely to permit greater flexibility and movement.

The CBD of σ^54^ is shown to interact with a number of key regions in RNAP and blocks the RNA exit channel. Thus, it is suggested that σ^54^ would need to move position or be released upon synthesis of a certain length of RNA (15–17 nt) prior to elongation. Kinetic studies indeed showed that σ^54^ is released from polymerase upon elongation [39]. This is potentially in contrast with σ^70^, in which a significant fraction (∼ 30%) of elongation complexes were shown to retain σ^70^ during elongation [40], [41], [42], [43]. Interestingly, the CBD also interacts with the C-terminal domain of the RNAP alpha subunit (α-CTD) in the x-ray structure of the RNAP-σ^54^ holoenzyme (PDB ID: 5NWT [16]) and RPo (PDB ID: 6GH5 [18]). However, there is no clear density for α-CTD in the other complexes such as RPc (PDB ID: 5NSR), the intermediate complex RPi (PDB ID: 5NSS), the partially loaded complex RPip (PDB ID: 6GH6), or the initial transcribing complex RPitc (PDB ID: 6GFW), suggesting that this interaction is transient and dynamic. α-CTD has been shown to play many key roles, including promoter recognition through interactions with activator, repressor proteins and DNA, as well as coupling mRNA from RNAP to ribosomes during transcription–translation coupling [44], [45], [46]. There is no evidence so far that α-CTD interacts with bEBPs, but whether α-CTD and CBD of σ^54^ might play a coordination role in mRNA transfer is an intriguing idea that requires further investigation.

In summary, the σ^54^ domains block the DNA entry to the active site and occupy the downstream DNA channel, as well as the T-strand DNA and RNA exit channels, thus explaining the tight inhibition σ^54^ imposes on RNAP. In order for transcription to proceed to the open complex, for RNA synthesis and elongation, these domains need to be relocated. However, these domains are topologically constrained with polypeptide chains snaking back and forth. RI interacts with RIII-ELH. In between RI and RIII-ELH is RII.1, which occupies the downstream DNA site (Fig. 2d) and is connected to RII.2 and RII.3, embedded well into the DNA/RNA binding channel before exiting through the RNA exit channel to connect to the CBD, which then snakes back along a shallow groove to connect to RIII-ELH (Fig. 2e). The relocation of these several domains is thus unlikely to be a frequent spontaneous process, instead requiring substantial coordinated conformational changes within σ^54^ and RNAP, presumably driven by AAA + activator proteins.

## Mechanisms of σ^54^-Dependent Transcription Initiation

Activator proteins utilize their ATPase activities to remodel the stable closed complex and assist isomerization into the RPo state. The isomerization process is common to all transcriptional systems and is highly dynamic, involving short-lived intermediate states that are difficult to capture. Using the σ^54^ system and the activator protein trapped by an ATP transition state analogue, along with the advanced technology of cryoEM in separating structural states, a number of functional states, including two intermediates, have been captured. These structures now provide a detailed molecular basis for σ^54^-dependent transcription initiation with some aspects that are likely to be shared with other systems.

### Initial DNA separation and transcription bubble formation

A recent cryoEM structure of RPc reveals the mode of promoter recognition by σ^54^. Following RNAP recruitment, the DNA is bound at the two consensus sites, − 24 and − 12, by the RpoN box and RIII-HTH, respectively [17]. Significant distortions in DNA were observed downstream of − 12. Specifically, the minor groove around − 12 is widened significantly. This correlates with the positioning of the alpha helical RI of σ^54^, which contacts both DNA strands downstream of − 12, alongside the RIII-HTH domain which interacts with the non-template strand [17]. The structural constraints between RI and RIII-HTH, which is connected by the ELH, thus contribute to the widening of the DNA minor groove [18].

Although the DNA distortion and opening are initiated in RPc, an activator is required for further transcription bubble opening, as σ^54^ mutants that bypass the requirement of an activator can only initiate transcription with a pre-opened transcription bubble [32], [47]. In order to understand how the activator is engaged in DNA opening and assists the release of the σ^54^-inhibition on the RNAP holoenzyme, structures of activator bound RNAP-σ^54^–DNA complexes are required. Using cryoEM, and the ATPase domain of activator PspF (PspF_1–275_), which is sufficient to activate transcription in vitro [48], a structure of PspF_1–275_ trapped with ADP.AlF_x_ in complex with RNAP-σ^54^–promoter DNA, termed RPi, was obtained [17]. In support of previous data, it is evident from the cryoEM density that the interactions between the asymmetric PspF hexamer and σ^54^ are via two surface loops (L1 and L2 loops) of PspF and RI of σ^54^ [17], [38], [49], [50], [51]. Indeed, the interactions between RI and the L1/L2 loops form a wedge at − 12/− 11 and separates ∼ 5–6 bp, thus committing and promoting strand separation and transcription bubble formation [17]. Furthermore, the RI and RIII-ELH domains, which occupy the entry site for DNA into the RNAP cleft in RPc, have moved upstream in RPi, thus partially removing the inhibitory effect of σ^54^ on DNA loading. This is consistent with data showing that the RPi state can support short transcript synthesis using a dinucleotide primed RNA with a pre-opened transcription bubble template [52]. RI and RIII-ELH thus act as a retractable gate for DNA loading [17]. This movement also coincides with a widening of the RNAP cleft, again in preparation for DNA entry into the RNAP cleft.

### Initial DNA loading

In both RPc and RPo, the clamps are in a closed conformation, unable to accommodate a double-stranded DNA. However, in the intermediate complex RPi, the β′ clamp opens up, signifying the importance of capturing intermediate states of transcription initiation complexes in order to understand the mechanisms of the dynamic transcription initiation. It is clear from these structures that further conformational changes are required for DNA loading and to fully open the transcription bubble. A recent structure of another intermediate complex, when DNA is partially loaded into the RNAP cleft, shed light into these processes [18].

In the process of obtaining the open complex (RPo) structure, which was formed by using a σ^54^ activator-bypass mutant (R336A) along with a preformed transcription bubble containing a mismatch between − 10 and − 1 on the NT strand, and the initial transcribing complex (RPitc) structure, which was formed by adding a dinucleotide primer and selected nucleotides to the open complex, another conformational state was obtained and solved using cryoEM to 4.1-Å resolution.

**Fig. 3:**
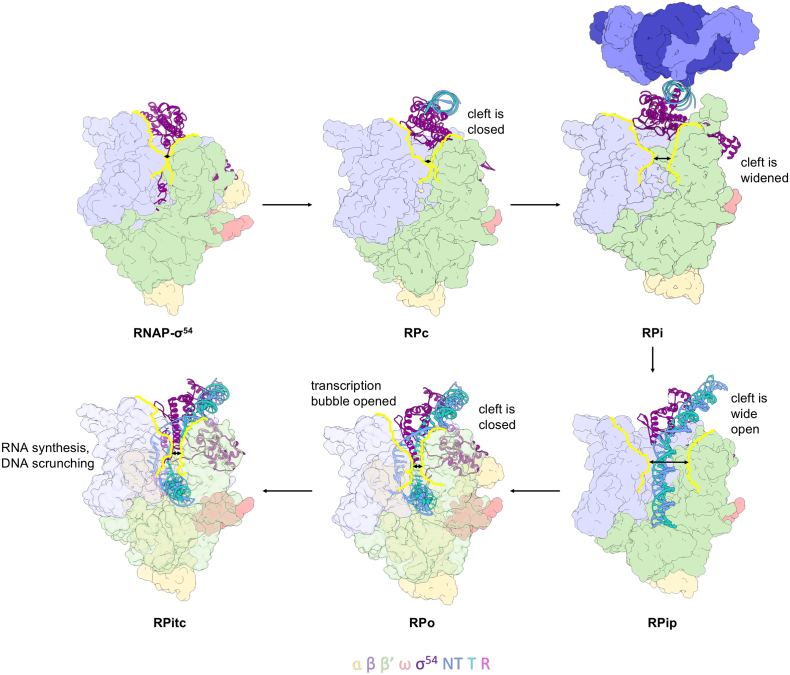
The known states of σ^54^-mediated bacterial transcription. Arrows indicate the cleft width at each stage, with the cleft outlined in yellow. Images were constructed using Chimera v1.9 with RNAP-σ^54^ (PDB ID: 5NWT), RPc (PDB ID: 5NSR), RPi (PDB ID: 5NSS), RPip (PDB ID: 6GH6), RPo (PDB ID: 6GH5) and RPitc (PDB ID: 6GFW).

In this structure, DNA was held at the RNAP cleft instead of being fully inserted, as expected in RPo or RPitc [18]. Instead, DNA exhibits a 30° kink toward the RNAP cleft at the − 12/− 11 position, signifying the point at which DNA may turn and enter into the active site during loading [18]. The β′ clamp is wide open in this structure compared to RPc (∼ 22 Å/20^o^ rotation), allowing loading of the dsDNA into the cleft (Fig. 3). Importantly, the DNA between − 10 and + 1 is held by the proline/glycine loop of the β pincer at residues 372–375, along with the positively charged β′ coiled-coil loop between residues 305–325 on the opposing side of the clamp, thus stopping dsDNA from being fully loaded [18]. This structure was thus proposed to represent an intermediate state where dsDNA is partially loaded into the cleft and has been termed RPip (Fig. 3).

Interestingly, a range of DNA conformations have been captured for eukaryotic RNAPII closed complex, where the dsDNA sits closer to the RNAP cleft compared to the closed complex of RNAP-σ^54^. In the human RNAPII closed complex structure, the DNA has a 10^o^ kink around − 12/− 11 toward the RNAP channel. This is accompanied by a slightly more open clamp [53], [54]. Recent work on the yeast RNAP II closed complex reveals a possible conformation with slightly distorted DNA [55]. The trajectory of DNA toward the RNAP cleft correlates with the widening of the cleft, thus supporting a model that initial DNA loading is accompanied by the clamp opening. The largest opening was captured in RPip, which correlates with DNA being furthest into the cleft. Although it cannot be ruled out that RPip is a collapsed RPo or RPitc state or an off-pathway intermediate, a number of arguments support the view that RPip might be an on-pathway intermediate. First of all, the RPip structural features agree with earlier DNA footprinting studies of RNAP-σ^70^ intermediate state I_1_ [56], [57]. Second, the clamp conformation and DNA path (including the 30^o^ kink) in the RPip structure are in agreement with the conformational trajectory based on those observed in RNAPII. Third, the wide opening of the clamp, as in RPip, was observed in RNAP clamp conformations using fluorescence resonance energy transfer experiments [58], which revealed a distribution of several conformational states for holoenzymes, although only closed clamps were observed in RPo. However, further investigation is required to correlate the exact clamp conformations in solution with the functional and structural states during initiation.

### A coupled load and unwind model

How DNA is loaded and unwound is at the core of the transcription initiation process and has been extensively studied. Earlier footprinting data indicated protected regions within the promoter, implying interacting sites with proteins or regions enclosed by proteins. Specifically, the interactions made at − 12 by σ^54^ were shown to be important in DNA distortion in the closed holoenzyme complex [59]. Potassium permanganate probing, combined with ortho-copper phenanthroline and diethylpyrocarbonate footprints, which preferentially interact with thymines on single-stranded DNA, was used to identify the selective interaction of σ^54^ with the single-stranded T-strand at this − 12 proximal promoter region [59]. These data first indicated a role for σ^54^ in transcription bubble initiation/stabilization.

Two intermediate states preceding the open complex of the σ^70^-RNAP holoenzyme, named I_1_ and I_2_, were also identified with footprinting and low-temperature equilibrium kinetic studies [56]. Analysis of both states suggests that binding of the DNA in I_1_ triggers a conformational change in the RNAP β and β′ jaws, which in turn results in their closure in I_2_, thus leading to opening of the DNA [56]. In I_1_, a sharp 90° kink in the DNA at − 10 is proposed in order to permit RNAP–DNA contacts downstream of − 5, likely to guide DNA into the active site cleft [57]. It is possible that the I_1_ state derived from early footprinting in the σ^70^ system relates to RPip in the σ^54^ holoenzyme complex. A 90° bend in DNA was detected in the I_1_ state, while a 30° bend was observed in σ^54^. This could reflect the different paths of their upstream promoter regions (see the Comparisons with σ70 section and Fig. 5c). I_2_ is a short-lived state where DNA is opened up, but not yet stabilized by RNAP as observed in RPo [18], [56]. Time-resolved footprinting demonstrated that the entire transcription bubble between − 11 and + 2 was opened up in a single step between I_1_ and I_2_ [60].

The molecular details on how DNA is finally loaded and the transcription bubble opened up have recently been proposed by comparing the structures of RPip and RPo, where DNA is opened up and the T-strand stably delivered into the active site for RNA synthesis. Interestingly, like RPo for σ^70^ and eukaryotic RNAPs, the clamp in σ^54^-dependent RPo is also in a closed conformation [18], [53], [54], [61]. The transition between RPip to RPo occurs by a 22° rotation of the β′ clamp, pivoted around the base of the clamp, resulting in a downward push of the clamp toward the active site. Double-stranded DNA is held in place upstream by the RpoN, RI and ELH-HTH domains of σ^54^ and downstream by the RNAP β′ jaw. The downward push of the β′ coiled-coil, with which DNA strands interact, would force the DNA strands to unwind, while it is being loaded into the active cleft due to the structural constraints imposed.

The data presented thus support a “coupled load and unwind” model of transcription initiation. The “coupled load and unwind” model suggests that loading of the dsDNA into the cleft prompts the transcription bubble opening in a single step, in support of previous kinetic data showing that the transcription bubble is opened up in a single step between I_1_ and I_2_ [60]. As σ^70^ spontaneously isomerizes, it has not yet been possible to capture a range of intermediate states to directly visualize the order of the DNA opening and loading events. Recently, a partially opened transcription bubble (between − 11 and − 4) was captured using *Mycobacterium tuberculosis* RNAP and a special AP3 promoter [62]. In this structure, the DNA is loaded into the cleft and the transcription bubble is not fully opened yet. But unlike in RPo, the cleft is not fully closed. It was suggested that this state was stabilized by the narrowing cleft between the β fork loop 2 (FL2) and β′ switch region 2 (SW2) and as such that the clamp must open for the final loading/melting stage before it is closed down again. This structure was thus suggested to be a true intermediate state due to intrinsic structural barriers within RNAP, representing I_2_ [61]. Interestingly, the AP3 promoter is GC rich in the transcription bubble region and indeed the transcription bubble captured in the structure stopped at A/T followed by three G/C pairs. It is thus not possible to rule out that the state captured is due to the promoter melting energetics of AP3. Indeed, promoter energetics play key roles in DNA melting as shown in the genome-wide analysis of promoter sequences in yeast systems [55]. Interestingly. when the *M. tuberculosis* RNAP structure is superimposed on RPo, clamp closure also causes an upstream movement of the clamp and thus could pull the downstream DNA into the RNAP channel, helping with the DNA melting. It is thus plausible that although this state does not necessarily represent a general intermediate state, this structure could represent a conformation *en route* from I_1_ (or RPip) to RPo, captured here by the use of the AP3 promoter. This structure is thus not inconsistent with the coupled load and melt model whereby the clamp closure causes the final loading and melting of the DNA (Fig. 3).

### A multi-step transcription initiation process

In summary, recent structural data suggest that transcription bubble formation and DNA loading in σ^54^-dependent transcription initiation can be divided into a number of distinct steps: (1) DNA distortion and initiation of strand separation/weakening, which occurs in RPc in the σ^54^ system: Kinetic data on the σ^54^ system are also suggestive of the existence of two closed complex states, with the second one being more stable, possibly due to DNA distortion and melting at − 11. This state is consistent with the closed complex structure captured with cryoEM [17], [39]. Whether this step occurs in other systems awaits further studies that are able to capture RPc. (2) Further strand separation and weakening, observed in RPi and specific for the σ^54^ system, could be due to the highly stable nature of σ^54^ promoters. This step also involves the release of the inhibition imposed by σ^54^. (3) Initial loading of DNA into the cleft: This involves a large opening of the cleft and DNA is partially loaded and stabilized by interactions with β′ coiled-coil loops, as in RPip. This step also involves partial unwinding of the dsDNA when it kinks at − 12/− 11. (4) The coupled load and unwind step: This step involves the closure of the cleft, which leads to the loading of the DNA into the cleft as well as melting out the DNA, completing the loading and unwinding process. (5) Finally, the transcription bubble is stabilized by RNAP interactions as observed in RPo. It is plausible that other transcription systems utilize similar mechanisms for the latter steps and recent work on RNAP II are in agreement with this model [55], [61].

What drives these conformational changes? Earlier steps are driven by σ^54^ and activator interactions, but once the inhibition is released, the conformational changes could be driven by the intrinsic clamp dynamics as fluorescence resonance energy transfer studies have shown the clamp to be highly dynamic [58], [63], [64]. Alternatively interactions between RNAP, σ and DNA might drive the conformational changes. For example, once the activator protein removes the inhibition imposed by RI of σ^54^, σ^54^ relocates, leading to new structural arrangements of the complex. Initially, clamp opening leads to initial DNA loading, which is then paused by interactions between DNA and β/β′, as captured in RPip. Subsequent clamp closure leads to the final DNA loading and melting (coupled load and unwind model).

## Roles of Activator Proteins

**Fig. 4:**
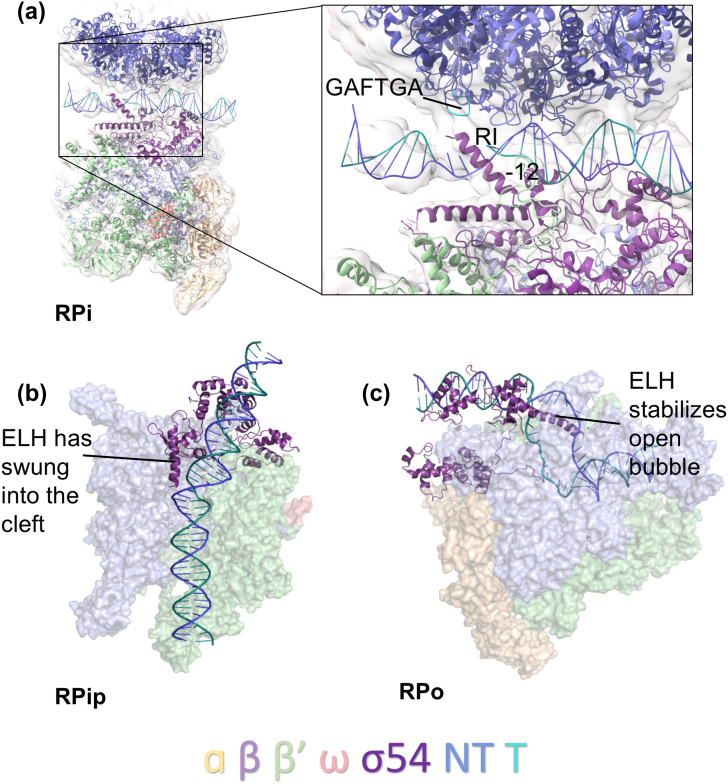
The unique features of σ^54^. (a) Density showing the interaction between RI of σ^54^ and the GAFTGA (cyan) sequence on L1 in RPi (PDB ID: 5NSS) using ChimeraX v0.1. (b) The ELH swings into the cleft, resulting in a kink in the DNA of 30° in RPip (PDB ID: 6GH6). (c) The ELH stabilizes the open bubble in RPo (PDB ID: 6GH5).

The ATPase activity of bEBPs is essential for isomerization of the RNAP-σ^54^ closed complex into the open complex [65]. The conserved GAFTGA signature motifs of bEBPs are within L1 of the AAA + domain and are shown to contact RI of σ^54^ (Fig. 4a), conformationally coupling the ATPase domain of the bEBP to the σ^54^ complex [66], [67], [68], [69]. In particular, the central threonine residue at position 86 in PspF L1 was identified as an important component for the detection of the DNA conformation downstream of − 12, playing a major role during energy coupling [47], [66], [70]. In fact, each of the six residues within this motif has been identified as essential for RPo formation through mutagenesis studies within a variety of bEBPs including PspF, NtrC, NifA, DctD and DmpR, the effects of which are well summarized in Bush and Dixon [8]. In particular, the phenylalanine residue has been consistently identified as essential for transcription activation, although in a small number of instances, a tyrosine residue is present at this position instead, suggestive of a role for aromatic rings in transcription activation [8].

The exact interactions that occur between the L1/L2 loops and RI have been characterized using cryoEM and small-angle x-ray scattering for a number of activator proteins including NtrC and PspF [51], [68], [69]. A 1.75-Å resolution crystal structure of PspF_1–275_ showed that the L1 and L2 loops protrude from the AAA + α/β sub-domain [51]. This structure was compared to that of NtrC1, a bEBP from *Aquifex aeolicus* with 47% similarity to PspF's AAA + domain, which revealed a number of differences in loop positioning. In the 3.1-Å crystal structure of ADP-bound NtrC1 (PDB ID: 1NY6), the L1 loop protrudes into the lumen of the heptamer, with a hydrophobic cluster of residues (L263, F216 and A206) positioned to lock the GAFTGA in a buried conformation, which is therefore most likely unable to form a stable interaction with σ^54^ [71]. These hydrophobic interactions are disrupted within ATP-bound PspF. The L1 and L2 become flexible and are hypothesized to be in a conformation amenable for engaging with σ^54^ [48], [51]. Detailed comparisons of structures of PspF_1–275_ in complex with ATP and ADP showed that the Walker B motif residue E108 adapts different conformations in a nucleotide dependent manner and that this change is sensed by N64 and subsequently relayed to the L1 and L2 loops [48], [72]. The E108–N64 pair, conserved in most AAA + proteins, is termed the glutamate switch and is proposed to link substrate binding sites with nucleotides [73].

More recently, the cryoEM structure of RPi at 5.8 Å revealed valuable details regarding the role of the GAFTGA region of PspF. The density corresponding to hexameric PspF was clearly oriented above the DNA, with both L1 and L2 not only facing the DNA and RNAP-σ^54^, but also interacting with DNA [17]. The σ^54^ RI is in close proximity to L1/L2 and DNA [17], in support of previous data that identified RI as the main contact site for bEBPs [38]. The electron density map indicates a connection between RI and the two loops, forming a wedge-like structure downstream of − 11/− 12 promoter site, proposed to stabilize DNA strand separation during transcription initiation [17]. These interactions also cause a slight relocation of RI and RIII-ELH from its positions in RPc (Fig. 4a). The glutamate switch model strongly suggests that nucleotide hydrolysis-driven conformational changes are propagated through these loop regions, which would in turn relocate RI, thus removing RI's inhibitory effects on isomerization.

The roles of activator proteins in relieving the inhibition placed on transcription by σ^54^ are predicted to be 3-fold. Initially, as indicated with the activator bypass mutants, which only permit transcription upon addition of a preformed bubble [32], bEBPs are required to stimulate DNA melting [17]. This is achieved by the interactions of L1/L2 with RI forming a wedge in separating DNA strands. Second, the activator must remodel the σ^54^ RI in order to remove the blockage within the RNAP β–β′ cleft that prevents DNA entry [16], [17], [38]. This is accompanied by the relocation of RI and RIII-ELH. Finally, DNA must be moved into the active site of RNAP. RI and RIII-ELH act as a rigid crowbar linking the β and β′ subunits. Removal of RI by the activator protein thus releases these constraints, permitting the opening of the cleft required for DNA entry. It is worth noting that bEBP subunits do not obey 6-fold symmetry, in agreement with the NtrC1 crystal structure [74]. Importantly, bEBP subunits directly interact with DNA and make extensive interactions with phospho-backbones between − 20 and − 12. Furthermore, strand separation is stabilized by the interaction between the L1/L2 loops and RI of σ^54^. Apart from forming a direct interaction with RI, the L1/L2 loops, especially the highly conserved GAFTGA motif, are also positioned to directly interact with DNA bases. However, higher-resolution structural information is required to precisely dissect the exact roles of these features of the transcription complex as well the nucleotide requirement and actions of individual subunits within the hexamer.

## Comparisons with σ^70^

σ^70^ and σ^54^ differ in a number of ways, with the most notable and important difference being the former's ability to spontaneously isomerize in the absence of an activator protein. Thus, σ^70^ activators largely act to increase the association of RNAP with the promoter region via interaction with promoter DNA and with the α-CTD of RNAP [75], [76]. In addition, σ^70^ recognizes − 35 and − 10 consensus sequences, whereas σ^54^ recognizes − 24 and − 12.

**Fig. 5:**
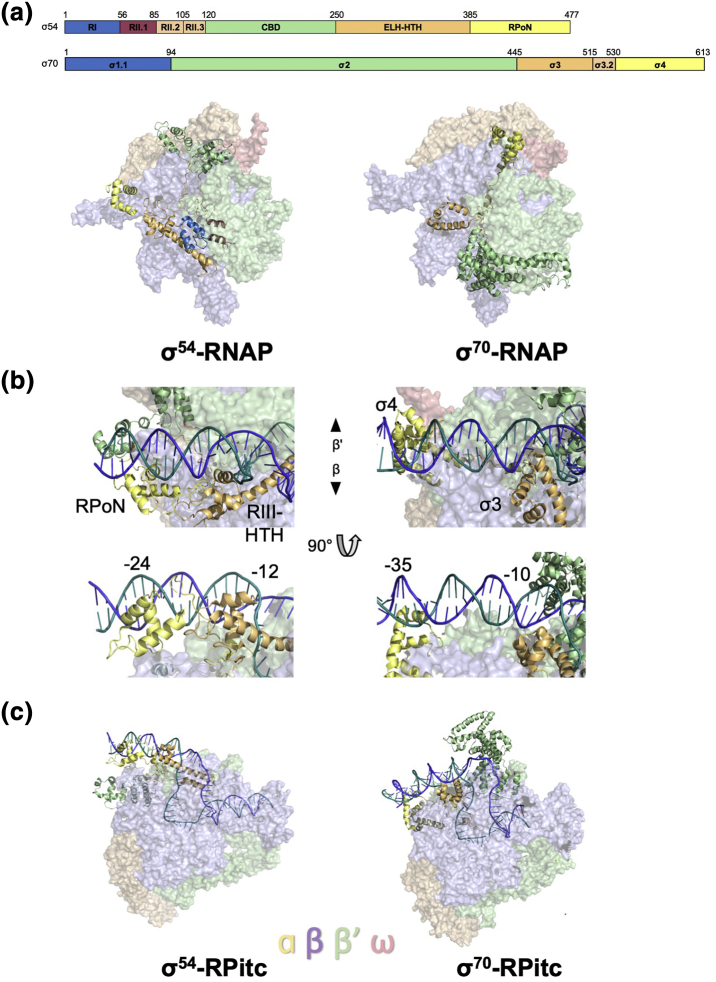
Comparison of σ^54^ features to σ^70^. (a), the four domains of each σ factor contact similar sites within the RNAP, despite the variations in their relative location. (b), DNA binding domain differences. (c), The open complexes show similar transcription bubbles, even though the upstream DNA paths vary. Images were created in PyMOL using σ^54^-RNAP (PDB ID: 5BYH), σ^70^-RNAP (PDB ID: 6C9Y), σ^54^-RPitc (PDB ID: 6GFW), and σ^70^-RPitc (PDB ID: 4YLN).

Sequence analysis combined with functional and structural studies has revealed that σ^70^ consists of four main domains, 1–4, in which region 4 contacts the − 35 promoter site and region 2.4 the − 10 site [5], [77]. Despite the lack of sequence conservation to σ^54^, these four domains contact similar sites on RNAP [16], [78] (Fig. 5a). However, the equivalent functional domains of σ^70^ and σ^54^ are located completely differently on RNAP. σ^70^ region 1.1 (residues 1–94) has been proposed to play an inhibitory role and thus is functionally similar to RI of σ^54^ [6]. However, structurally it is located similarly to RII.1 of σ^54^, which has high-sequence variability [16]. RI of σ^54^ forms a structural domain with RIII-ELH and interacts with the β′ subunit, located broadly similarly to region 2 of σ^70^, which is the main RNAP interaction domain. On the other hand, the main RNAP binding domain, CBD of σ^54^, is located at the β′ side blocking RNA exit. Interestingly, this region of RNAP is occupied by region 4 of σ^70^, thus contacting − 35 on the β′ side, as opposed to the RpoN box, which is located on the β side contacting the − 24 region [16], [18]. These differences in DNA binding domains confer the different promoter recognitions, especially the spacing between conserved σ DNA recognition elements and define the different promoter paths (Fig. 5b). Region 3 of σ^70^, which is in close proximity to the − 10 consensus sequence, is located on the β side, similarly to RIII-HTH of σ^54^, which interacts with the − 12 region (Fig. 5b). As a result, the − 12/− 10 site of promoter DNA is brought within close proximity within both σ^54^ and σ^70^ complexes. This is not surprising as the transcription bubbles for both the σ^54^ and σ^70^ systems are almost identical in the open complexes, both consisting of ∼ 15 nt (− 12 to + 3), which can be overlaid despite the different upstream promoter paths (Fig. 5c) [18].

As mentioned above, one of the key differences between the two systems is σ^70^'s ability to spontaneously isomerize into an open complex. Within region 2.3 are a number of highly conserved Trp residues that interact with the non-template strand and stabilize the opened bubble [79]. As a result, base flipping that is subsequently stabilized by these Trp residues has been proposed to be key in nucleating DNA melting and transcription bubble stabilization [27], [79]. Within σ^54^, RI and RIII-ELH have a limited number of conserved aromatic residues, and therefore, it was hypothesized and since demonstrated that promoter melting requires bEBP activity, as discussed above.

Sequence similarity has been observed between RII.3 of σ^54^ and region 3.2 of σ^70^, both of which have been shown to occupy the RNA exit channel and have a number of acidic residues proposed to guide RNA exit [16], with the latter also involved in initial transcription pausing [80], [81]. DNA bases are flipped at − 4/− 3 in order to contact these residues, and when the 5′ triphosphate end of RNA reaches the acidic residues of this region, the tip of region 3.2 becomes disordered and is eventually pushed out as the RNA extends [82]. As a result of their homology, it is hypothesized that RII.3 may impart a similar function.

## Unique Features of σ^54^ Define Its Functions

Why is σ^70^ able to spontaneously isomerize, whereas σ^54^ cannot? There are a number of reasons, of which many have been discussed above. The coupled load and unwind model suggests that clamp closure is sufficient to load and unwind the transcription bubble. However, this is only sufficient for those promoters that are easy to melt, thus having a lower energy barrier to overcome for strand separation. In fact, recently it has also been shown in RNAPII that mutating promoter regions can bypass the requirement of TFIIH, the eukaryotic ATPase required for transcription initiation, supporting the idea that intrinsic clamp dynamics in RNAP are sufficient to drive promoter opening up to + 1, provided the promoter has a low-energy barrier to melt [55].

We thus focus here on the differences of σ^54^ and σ^70^ in terms of their abilities to melt promoter DNA and in stabilizing the transcription bubble. In the absence of structural information on the closed and intermediate complexes for σ^70^, some of the comparisons are speculative. (1) First of all, σ^70^ is proposed to use the aromatic Trp residues to initiate and stabilize a flipped out base at the upstream transcription bubble around − 12/− 11 [27], [79]. σ^54^ lacks equivalent conserved aromatic residues in RI and RIII-ELH, which are proximal to the − 12 region. In fact, structural data show that initial DNA distortion occurs in RPc but is unable to proceed further. Activator proteins are required to further initiate formation of the transcription bubble. Work so far suggests that activator proteins actively promote strand separation by opening up to 5–6 base pairs (Fig. 4a) [17]. (2) Second, apart from the structural features in σ^54^ that prohibit DNA melting, it is also highly likely that some σ^54^ promoters are more difficult to spontaneously melt, similarly to those recently reported in RNAP II [55]; thus, some activator assisted pre-opening is required. (3) Third, σ^70^ initiates the strand separation in cohort with region 2 and region 3, arranged in a V-shape (Fig. 5c), to stabilize the two strands, thus capturing and stabilizing partially opened segments, which can occur more readily. σ^54^, on the other hand, uses a single structural element, the ELH, which swings into the cleft before separating the two strands (Fig. 4b and c). An extensively opened-up section in the promoter DNA is thus required in order for the ELH to stably insert and separate the two strands. This process thus has a significantly higher-energy barrier, which speaks to the involvement of ATP in driving the conformational changes needed for the isomerization step. (4) Finally, our current understanding shows that conformational changes in RNAP play key roles in DNA loading and unwinding. σ^70^ region 2 and region 3, linked by a flexible linker, interact with β and β′ subunits separately and thus permit the large conformational changes required in RNAP (Fig. 5a). σ^54^, on the other hand, when RI is present, contains a rigid crowbar formed by RI and ELH between β and β′ (Figs. 2c and 5a), thus limiting conformational flexibility, blocking DNA loading and unwinding.

## Future Perspective

Our understanding of σ^54^-dependent transcription has seen a significant advance over the last few years with the availability of structures for the holoenzyme, the closed and activator-bound intermediate complexes, the partially loaded intermediate complex and the open and the initial transcribing complex. This set of structural data, combined with earlier structural data on the activator proteins as well as biochemical and kinetic data, has provided significant insights into how the transcription bubble opens, DNA is loaded, and RNA is synthesized. Furthermore, we also have a wealth of information on how σ^54^ inhibits transcription and how activator proteins act to first relieve this inhibition and then drive DNA opening. However, these structures only capture snapshots and are under conditions that facilitate trapping of intermediates (such as pre-melted bubbles and nucleotide analogues). It is therefore imperative that further studies using wild-type promoters that are driven by activators are employed to corroborate these findings. More importantly, real-time single-molecule kinetic studies are extremely powerful in elucidating transition state kinetics, such as those shown for pausing and initial transcription for σ^70^ system [80], [81]. Similar studies will be invaluable to capture the transition kinetics in different promoter and holoenzyme complexes, as well as to identify multiple reaction pathways and their respective on and off-pathway species, in order to fully extract mechanistic insights. For σ^54^-dependent transcription, we still do not know exactly how nucleotide binding and hydrolysis drive activator-mediated conformational changes within the hexamer and the particular molecular details involved in the relocation of RI and driving of DNA melting by these activators. Furthermore, we do not know how activator-bound upstream DNA is engaged within the transcription complexes as they isomerize and, in many cases, how activator proteins are regulated by upstream signals. To further develop our understanding of these mechanisms, additional structural, kinetic and functional studies are required in order to capture meaningful functional complexes.
